# The role of 3D speckle tracking echocardiography in the diagnosis of obstructive sleep apnea and its severity

**DOI:** 10.1038/s41598-022-26940-2

**Published:** 2022-12-26

**Authors:** Ioana Maria Chetan, Bianca Gergely-Domokos, Ruxandra Beyer, Raluca Tomoaia, Georgiana Cabau, Damiana Vulturar, Ana Chis, Andrei Lesan, Cristian Stefan Vesa, Dana Pop, Doina Adina Todea

**Affiliations:** 1 Department of Pneumology, “Iuliu Hatieganu” University of Medicine and Pharmacy, Cluj-Napoca, Romania; 2 Heart Institute “Nicolae Stancioiu”, Cluj-Napoca, Romania; 3 Department of Cardiology, “Iuliu Hatieganu” University of Medicine and Pharmacy, Cluj-Napoca, Romania; 4 Department of Medical Genetics, “Iuliu Haţieganu” University of Medicine and Pharmacy, Cluj-Napoca, Romania; 5 Department of Pharmacology, Toxicology and Clinical Pharmacology, “Iuliu Hatieganu” University of Medicine and Pharmacy, Cluj-Napoca, Romania

**Keywords:** Cardiology, Health care, Medical research

## Abstract

There is a consistent relationship between obstructive sleep apnea (OSA) and cardiovascular diseases. It is already recognized that OSA may influence the geometry and function of the right ventricle (RV). This has encouraged the development of echocardiographic evaluation for screening of OSA and its severity. Three-dimensional speckle tracking echocardiography (3D STE) is in assumption better, compared with 2D STE, because it overcomes the standard 2D echo limitations. Thus, the purpose of our study is to evaluate whether 3D STE measurements, could predict the positive diagnosis and severity of OSA. We enrolled 69 patients with OSA and 37 healthy volunteers who underwent a cardiorespiratory sleep study. 2DE was performed in all patients. RVEF and 3D RVGLS were measured by 3DSTE. NT pro BNP plasma level was also assessed in all participants. 3D RV GLS (− 13.5% vs. − 22.3%, *p* < 0.001) and 3D RVEF (31.9% vs. 50%, *p* < 0.001) were reduced in patients with OSA, compared with normal individuals. 3D Strain parameters showed better correlation to standard 2D variables, than 3D RVEF. Except for NT pro BNP (*p* = 0.059), all parameters served to distinguish between severe and mild-moderate cases of OSA. 3D STE may be a reliable and accurate method for predicting OSA. Consequently, 3D RV GLS is a good tool of assessing the RV global function in OSA, because it correlates well with other established measurements of RV systolic function. Furthermore, 3D RV GLS was a precise parameter in identifying severe cases of OSA, while NT pro BNP showed no association.

## Introduction

Obstructive sleep apnea (OSA) is one of the most common forms of sleep disorders, affecting approximately one billion people around the world^1^. It is characterized by repeated partial or complete obstructive events, leading to intermittent hypoxia, sleep fragmentation, snoring and daytime somnolence, even with an acceptable duration of sleep^2,3^. Obesity or anatomic features have traditionally been implicated as the primary factors driving the pathogenesis of OSA. Despite this, OSA can occur without obesity or clinically evident structural compromise of the upper airway. As a result, the pathogenesis of OSA varies from individual to individual. Physiologic studies of upper airway function in OSA have recognized several mechanisms of sleep-related upper airway instability. Among the key mechanisms underlying sleep-induced narrowing and occlusion of the upper airways are: the capacity of the upper airway dilator muscles to respond to respiratory challenge during sleep, upper airway anatomy, the tendency to wake from increased respiratory drive during sleep and the stability of the respiratory control system^4^.

Initial reports of OSA were of severe apnea in obese male patients, however it is now known that women account for one third of OSA patients and a normal body mass index (BMI) is frequently associated^5,6^. OSA patients are mainly clinically supervised by sleep physicians with work experience in respiratory medicine. Nevertheless, many patients are asymptomatic from their apneas, seeing a cardiologist when having arterial hypertension, atrial fibrillation, stroke, coronary artery disease or heart failure^7^.

Observational studies have shown a consistent relationship between OSA and cardiovascular diseases (CVD)^8,9^. Such an association is largely due to autonomic, inflammatory, hemodynamic and metabolic consequences of this obstructive sleep pattern. The effects may also contribute to the pathogenesis of a variety of cardiovascular diseases. Repetitive apneas expose cardiovascular system to cycles of hypoxia and excessive negative intrathoracic pressure. These mechanisms can, sequentially, depress myocardial contractility and parasympathetic activity, activate the sympathetic nervous system, raise blood pressure, heart rate, myocardial wall stress, induce oxidative stress, systemic inflammation, activate platelets and damage vascular endothelial function^10^. As has been demonstrated, OSA is a condition, that has the potential for negative feedback, because it worsens conditions that could in turn aggravate OSA^11^.

The suspicion of OSA is based on symptoms and confirmed with diagnostic testing. Polysomnography is the “gold standard” to diagnose and characterize the severity of OSA^12^. However, OSA is extensively underdiagnosed; up to 95% of people from population surveys with clinically notable OSA reveal no previous OSA diagnosis^13^. In a published statement, the U.S Preventive Services Task Force (USPSTF) concluded that the current evidence is insufficient to assess the balance of benefits and harms of screening for OSA in asymptomatic adults^14^. Despite this statement, the high cardiovascular (CV) morbidity and mortality associated with this disease, alongside with significant improvements in quality of life, mood and social life with OSA treatment, provide solid grounds for OSA screening.

Screening for OSA is usually performed using questionnaires, typically having high sensitivity but low specificity, which limits their value as screening tools^15^.

OSA may affect the function and geometry of the heart chamber, especially that of the right ventricle (RV). This has stimulated the interest in the development of echocardiographic methods for screening for OSA and assessing its severity^16^. RV has a complex structure, makingconventional two-dimensional (2D) echocardiography techniques insufficient for accurate assessment^17^. Lately, 2D speckle tracking echocardiography (STE), which detects subclinical RV dysfunction, has shown to be a reliable and feasible quantitative technique for the assessment of RV function^18,19^. Furthermore, there is evidence suggesting that RV longitudinal strain of free wall (RV FWLS) derived from 2D STE offers prognostic information over common RV parameters in different clinical scenarios, including patients with pulmonary hypertension (PHT), heart failure (HF) or coronavirus-19 disease^20,21^. This is however a technique with limitations, the main ones being the fact that it only evaluates deformation in a 2D plane, the views can be foreshortened, and geometric modeling is difficult. Moreover, 2D STE assessment is only obtainable in the apical four chamber view (A4C), thus excluding the evaluation of RV outflow sections^22^. Three-dimensional (3D) STE appears to be better, compared with 2D STE, because it assesses deformation in 3 orthogonal planes from one analysis, overcoming the previously mentioned limitations^23^.

Evaluation of RV strain using 3D STE has been shown to be feasible^24^. Results from a single study demonstrate the prognostic utility of 3DSTE of the RV (using a software designed for the LV) to predict mortality among patients with PHT^25^. The value of this novel technique for RV assessment in OSA patients has not yet been established.

The aim of this study is to investigate whether 3D STE measurements, compared with standard RV 2DE parameters, could predict the positive diagnosis and severity of OSA.

## Material and methods

### Study population

Between January 2020 and February 2021, we examined and screened for OSA a cohort of 163 adult patients admitted to “Leon Daniello” Pneumology Hospital of Cluj-Napoca. The study was conducted according to the guidelines of the Declaration of Helsinki. The approval of the Human Research Ethics Committee (number: 493/2019) was obtained and patients signed an informed consent.

Inclusion criteria for patients were age ≥ 18 years, and presence of minimum 3 clinical symptoms of OSA. The symptoms were snoring, witnessed apneas, gasping/choking episodes, excessive daytime sleepiness not explained by other factors, nocturia, morning headaches and decreased concentration and memory.

We excluded from the beginning patients with decompensated or unstable cardiopulmonary disease, chronic respiratory disease such as chronic obstructive pulmonary disease (COPD), idiopathic pulmonary fibrosis (IPF) and sarcoidosis, cor pulmonale, known history of pulmonary embolism, significant right and left-sided valve disease, known cardiomyopathies, congenital heart disease, pericardial disease, malignancy, psychiatric disorders or recent surgery.

**Figure 1:**
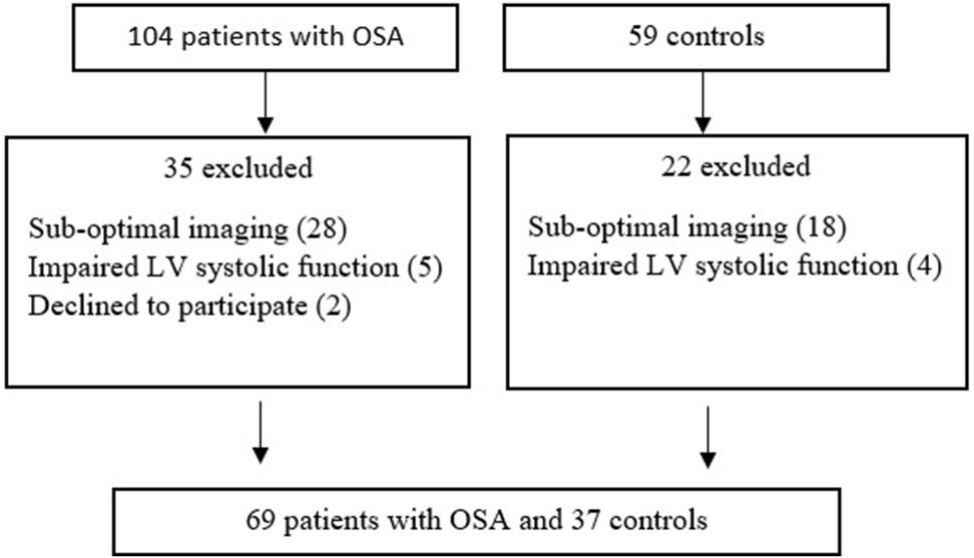
Study flowchart.

Finally, 106 participants were eligible for taking partin the study. They were divided into two groups; 69 patients were clinically diagnosed with OSA and they created the study group. The control group was formed by 37 sex and age-matched subjects, who, following respiratory polygraphy, did not have OSA criteria. At that time, the OSA patients were not under continuous positive airway pressure (CPAP) or bilevel positive airway pressure (BIPAP) treatment (Fig. 1).

### Sleep study

All enrolled participants (including healthy subjects) underwent cardiorespiratory sleep study using Nox T3 polygraphy device for a wholenight. The sleep study involved continuous recording from nasal cannulae, oxygen saturation, heart rate, tracheal sounds (microphone), thoracic and abdominal movement and body position. The results of the polygraphy were analyzed and approved by trained personnel. Apnea was defined as a complete cessation of inspiration for at least 10 s (s). Hypopnea was defined as a limiting in airflow by at least 50% for at least 10 s. Obstructive apnea was described as the absence of respiratory airflow in the presence of paradoxical thoracic and abdominal movement^26^. The number of apneas and hypopneas was defined as apnea–hypopnea index (AHI). OSA was diagnosed in the event that AHI was greater than or equal to 5, with recorded symptoms of daytime sleepiness, insomnia, involuntary sleep episodes during wakefulness, mood disorders, snoring, breathing disturbances or documented history of stroke, hypertension, ischemic heart disease. Patients with AHI between 5 and 15 are considered a mild form of OSA, patients with AHI values between 15 and 30 are counted as moderate forms and patients with AHI greater or equal to 30 are considered to have severe OSA^27^.

### Echocardiography

Echocardiographic data were obtained for all participants. All echocardiography images were acquired on a Vivid E95 scanner (GE Vingmed Ultrasound, Norway) and analyzed offline using EchoPac BT13 software (GE Vingmed Ultrasound, Norway). The following conventional RV echo measurements were performed using a 2D matrix-array (M5S) according to current recommendations^28^: basal right ventricle (RV) diameter in the apical four chamber view (A4C) at the end-diastole, tricuspid annular plane systolic excursion (TAPSE), S’ wave and fractional area change (FAC), as parameters of RV systolic function in a standard A4C; left ventricular ejection fraction (LVEF), mean tricuspid regurgitant gradient and pulmonary artery systolic pressure (SPAP) respectively. The LVEF was measured using manual tracing (biplane Simpson’s) method.

**Figure 2:**
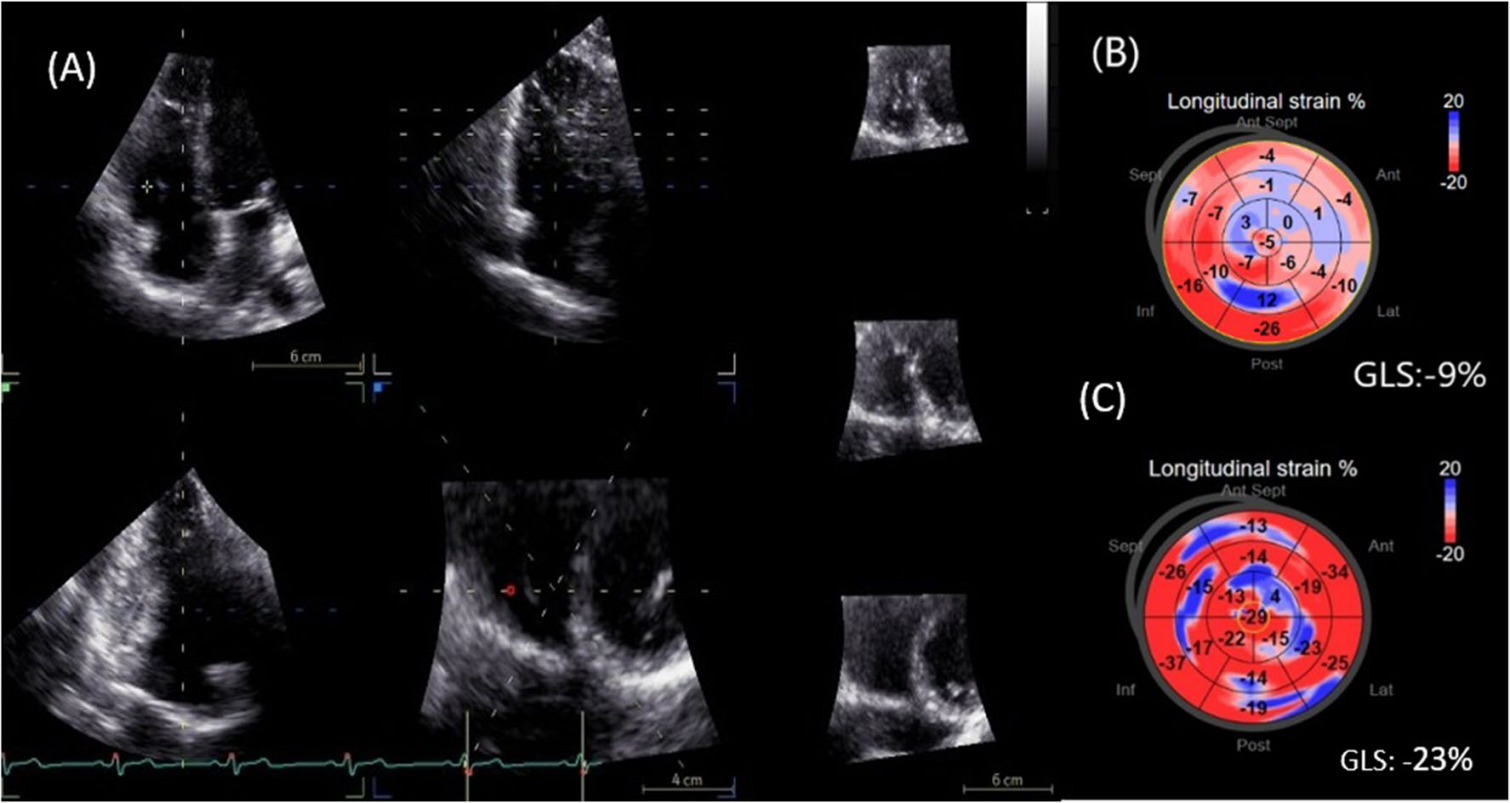
Representative RV 3D-STE analysis in two patients with OSA. (**A**) Automatic RV 3D-STE analysis using 4DAutoLVQ function. (**B**) Bull’s eyes reconstruction of 3D GLS in a patient with severe OSA ( decrease of GLS =  − 9%) respectively, in a healthy volunteer ( GLS =  − 23%). RV, right ventricle; 3D, three-dimensional; STE, speckle-tracking echocardiography; OSA, obstructive sleep apnea; GLS, global longitudinal strain.

By using a real time 3D phased-array transducer (4 V-D), 3D full volume data set of the RV over 4–6 cardiac cycles was recorded. The images were acquired in the apical window and aligned so that the entire RV could be integrated into the full volume dataset. The obtained 3D datasets were transferred to the EchoPac BT13 station for offline analysis. 3D RVEF was calculated using the 4D RV quantification function of the system (4D- AutoRVQ). 3D STE was performed using the GE 4D LV quantification function of the system (4D-autoLVQ), this software being adapted for the evaluation of RV since it was designed for the left ventricle STE analysis. After the three apical long axis and short axis view alignment, topographic landmarks were manually placed at the level of the tricuspid annulus and RV apex. The outline for the end-systolic and end-diastolic boundaries of the RV was automatically defined and manual adjustment was done where necessary. Accordingly, the automatic alignment of endocardial and epicardial contours was followed by manual correction, confirming the myocardial wall, where needed, in order for all segments to be included in the strain analysis. The software automatically calculated the global longitudinal strain (GLS) values. Bull’s eye diagrams, tracking curves were stored. (Fig. 2).

### Anthropometric and biochemical measurements

In all patients, fasting blood samples were drawn in order to evaluate levels of amino-terminal-pro-brain-natriuretic-peptide (NT-pro BNP). Plasma levels were measured by sandwich enzyme-linked immunosorbent assay (ELISA SK-1204, Biomedica Immunoassays, Vienna, Austria). Values were expressed as pg/mL. Body weight and height were measured and recorded. Body mass index (BMI) was calculated with the formula of body weight/height^2^ (kg/m^2^). The same person performed all the measurements using the same tools. Obesity was defined as a body mass index (BMI) ≥ 30 kg/m2.

### Interobserver variability

To evaluate the reproducibility of the 3D-STE measurements, we have randomly selected 20 patients in whom measurements were repeated by a second operator, who was blinded to the results of the first operator. We calculated an inter-observer correlation coefficient of 0.867.

### Statistics

Statistical analysis was performed using the MedCalc^®^ Statistical Software version 19.7 (MedCalc Software Ltd, Ostend, Belgium; https://www.medcalc.org; 2021). Quantitative data were examined for normality of distribution using the Shapiro–Wilk test and expressed as median and 25–75 percentiles. Qualitative data were expressed as frequency and percentage. Comparison between two groups for categorical variables was by chi-square test and by Mann–Whitney test for continuous variables. Correlation between variables was assessed by Spearman correlation coefficient. The sample size was calculated from the first 10 controls and 16 OSA patients. For the OSA patients we determined a 3D RV GLS of − 14.5 ± 5, and for controls a 3D RV GLS of − 20.6 ± 0.7. For a type 1 (a) error of 0.05 and a type 2 (b) error of 0.1, we calculated a sample size of 18 controls and 36 OSA patients. The sensitivity and specificity of strain vector and the others standard parameters cutoff points were settled by the basis value indicated by the Youden index from the receiver-operating characteristic (ROC) curve to predict the disease. A “*p*” value lower than 0.05 was considered statistically significant.

### Ethics approval

“The study was conducted according to the guidelines of the Declaration of Helsinki, and approved by Ethics Committee of the University of Medicine and Pharmacy “Iuliu Hațieganu”, Cluj-Napoca number 493 on 21.11.2019.

### Informed consent

Informed consent was obtained from all participants involved in the study.

## Results

### Baseline characteristics

The baseline characteristics for both groups are detailed in Table 1. There were no significant differences in prevalence by age and sex between the two groups. Median BMI for the study population was 34 (IQR 30.8; 40.2) in OSA patients and 29 (IQR 28.5; 30.1) in controls, with significant differences between the two groups, even though the BMI values in both groups indicate at least overweight.

**Table 1:** Baseline characteristics of randomized patients.

**Variable**	**Patients** (n = 69)	**Controls** (n = 37)	***P* value**
Age years	59 (51; 67)	55 (47; 62)	0.394
Female sex, n, %	31 (44.9%)	18 (48.6%)	0.871
BMI kg -m^2^	34 (30.8; 40.2)	29 (28.5; 30.1)	< 0.001
**Additional cardiovascular risk factors**			
DM type 2, n , %	11 (15.9%)	5 (13.5%)	0.96
Arterial hypertension, n, %	63 (91.3%)	22 (59.5%)	< 0.001
Smoking , n , %	20 (29%)	17 (45.9%)	0.125
**Results of the sleep study**			
AHI h^-1^	28.6 (18.3;42.1)	2.8 (1.2;3.3)	< 0.001
ODI h^-1^	29.2 (21.9; 42.9)	3.5 (2; 6.2)	< 0.001
**Echocardiographic parameters**			
RV, mm	40 (37;43)	36 (33;37)	< 0.001
TAPSE, mm	19 (17;20)	23 (21.5;24)	< 0.001
S’ wave, cm/s	9.6 (9;11)	12 ( 11.9; 13.5)	< 0.001
RA-RV Gradient, mmHg	35 (30; 40)	19 (16.5; 22.5)	< 0.001
sPAP, mmHg	42 (37; 47 )	22 (16.5; 24.5 )	< 0.001
RV FAC, %	28 (25; 30 )	35 (33.4;45)	< 0.001
3D RVEF, %	31.9 (27.8;39)	50 (45.4; 59)	< 0.001
3D RV GLS, %	− 13.5 (− 17.2; − 10.6)	− 22.3 (− 28.3;− 20.9)	< 0.001
LVEF, %	55 (50;55)	55 (55;63)	0.09
**Serum parameters**			
NT-pro BNP, pg/ml	290 (201;620)	35 (15;127.5)	< 0.001

Data are presented as median (25–75 percentiles) and n (%); BMI: body mass index, AHI: apnea–hypopnea index, ODI, overnight desaturation index, DM: diabetes mellitus, RV: right ventricle, TAPSE: Tricuspid annular plane systolic excursion, RA: right atrium, sPAP: Pulmonary artery systolic pressure, FAC: fractional area change, RVEF: right ventricle ejection fraction, GLS: global longitudinal strain, LVEF: left ventricle ejection fraction, NT-pro BNP: amino-terminal-pro-brain-natriuretic-peptide.

Posibile pulmonary hypertension as assessed by echocardiography, i.e. sPAP > 37 mmHg was present in 84.1% of OSA patients, respectively 15.9% of OSA patients did not have PHT. There was significant difference in NT pro BNP values between the two groups.

The healthy volunteers’ mean 3D global longitudinal strain of the RV was significantly higher than in patients with OSA. Similar results were also obtained for the 3D RVEF.

### 3D STE and conventional RV function parameters

The standard RV function parameters (TAPSE, FAC, S′ wave) correlated significantly with 3D RV GLS (Table 2). Furthermore, 3D RV GLS correlated better than 3D RVEF with previously mentioned parameters. There was also a strong correlation between 3D RV GLS and 3D RVEF and a good correlation between 3D RV GLS and NT pro BNP, as well as a significant correlation between 3D RVEF and NT pro BNP.

**Table 2:** Correlations of 3D RV GLS and 3D RVEF with conventional RV function parameters and NT pro BNP.

Variable	3D RV GLS		3D RVEF	
	*r* value	*P* value	*r* value	*P* value
3D RV GLS			− 0.756	< 0.001
RV	0.701	< 0.001	− 0.482	< 0.001
FAC	− 0.791	< 0.001	0.789	< 0.001
TAPSE	− 0.737	< 0.001	0.653	< 0.001
S’ wave	− 0.749	< 0.001	0.623	< 0.001
sPAP	0.822	< 0.001	-0.711	< 0.001
NT pro BNP	0.714	< 0.001	-0.622	< 0.001

*RV* right ventricle, *TAPSE* tricuspid annular plane systolic excursion, *sPAP* pulmonary artery systolic pressure, *FAC* fractional area change, *3D RVEF* three dimensional right ventricle ejection fraction, *GLS* global longitudinal strain, *S*′ wave: systolic wave prime; *NT-pro BNP* amino-terminal-pro-brain-natriuretic-peptide.

All echocardiographic parameters were entered into a ROC model to calculate a cut-off value for predicting the presence of OSA (Table 3). 3D RV GLS with a cutoff value of > − 19.2% and sPAP with a cutoff value of > 30 mmHg, were the best predictors of OSA, with a sensitivity of 91.3% and specificity of 100%, respectively 94.2% and 100%. The parameter less predictive of disease was S′ wave (AUC = 0.85). It is important to highlight that even if FAC showed the best correlation with 3D RV GLS, in the ROC model it did not have the strongest predictive capacity of the disease, proving that the two statistic tests have different interpretation and roles. In the ROC analyses for detection of OSA, the best cutoff value of NT pro BNP was > 136 pg/ml, with a sensitivity of 91.3% and specificity of 91.8% (AUC = 0.93), similar to the diagnostic performance of 3D RV GLS (AUC = 0.979).

**Table 3:** ROC cutoff values predicting OSA.

Variable	Cutoff	Sensitivity (%)	Specificity (%)	AUC ( 95% CI )
RV, mm	> 39	59.4	100	0.835 (0.75–0.9)
TAPSE, mm	≤ 20	84	89	0.92 (0.85–0.96)
S’ wave, cm/s	≤ 10.2	68.1	91.8	0.85 (0.76–0.91)
RA-RV Gradient, mmHg	> 29	82.6	94.5	0.94 (0.87–0.97)
sPAP, mmHg	> 30	94.2	100	0.98 (0.94–0.99)
RV FAC, %	≤ 31	79.7	91.8	0.9 (0.83–0.95)
3D RVEF, %	≤ 41	88.4	94.5	0.94(0.88–0.98)
3D RV GLS, %	> − 19.2	91.3	100	0.979 (0.93–0.99)
NT-proBNP, pg/ml	> 136	91.3	91.8	0.96 (0.9–0.99)

**Figure 3:**
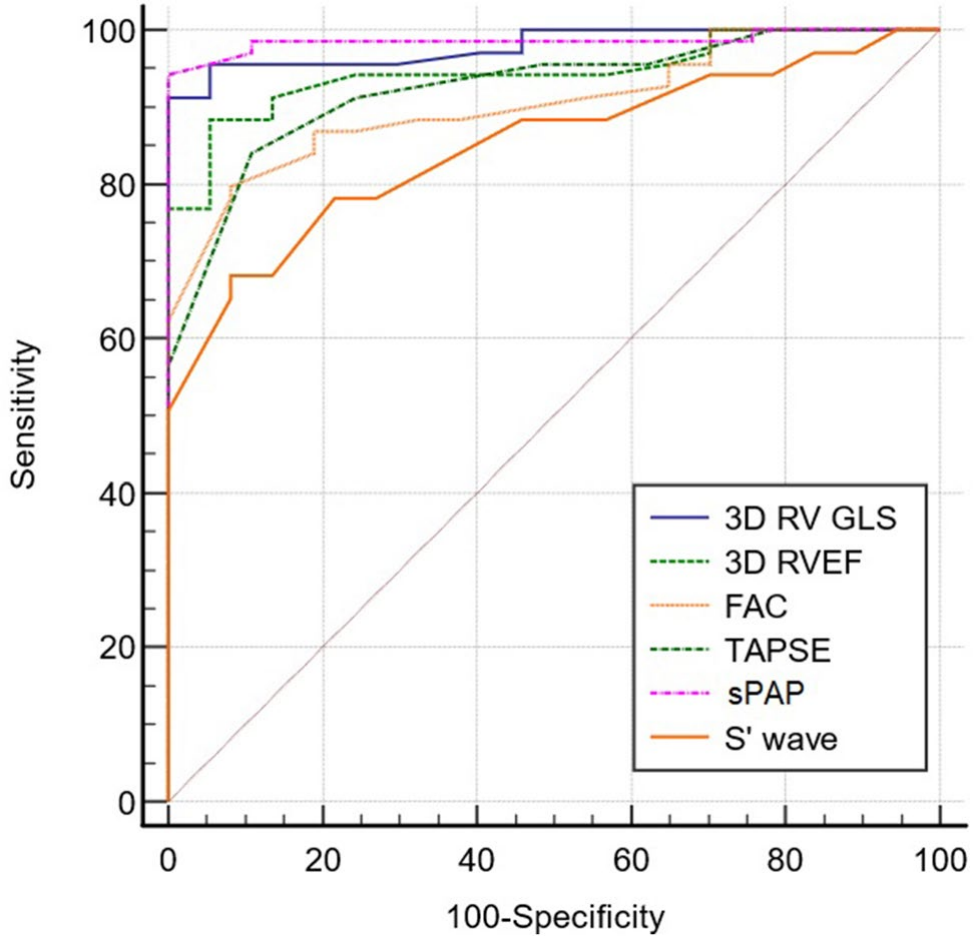
Comparison of ROC curves for prediction of OSA.

In order to evaluate which echocardiographic method has the ability to be the most discriminative regarding the positive diagnosis of OSA, comparison of ROC curves was calculated (Table 4, Fig. 3). The highest statistical significance was obtained when comparing the 3D RV GLS ROC curve with the ROC curve of S’ wave (*p* < 0.001), followed by TAPSE (*p* = 0.02). No significant differences between the other ROC curve comparisons were found.

**Table 4:** Comparison of ROC curves.

3DE parameters	2DE parameters	*P* value
3D RV GLS	RV FAC	0.005
3D RV GLS	TAPSE	0.02
3D RV GLS	S’ wave	< 0.001
3D RV GLS	sPAP	0.6
3D RVEF	RV FAC	0.1
3D RVEF	TAPSE	0.6
3D RVEF	S’ wave	0.001
3D RVEF	sPAP	0.09
3D RVEF–3D RV GLS		0.1

### Comparison between severe OSA patients versus non severe OSA patients according to 3D STE and standard 2DE

Based on the values of AHI, the study group was divided in two subgroups, as follows: severe OSA (AHI > 30) was present in 41 patients and non-severe OSA with AHI < 30 was present in 28 patients. All patients included in the severe group also presented PHT. When evaluating which echocardiographic method is better associated with severe OSA cases, FAC and sPAP obtained the highest statistical significance among 2DE parameters (*p* = 0.001). Additionally 3D RV GLS is maintaining the significant status amidst 3D STE evaluation (*p* = 0.01) in differentiating the severity of the disease. Concerning the capacity in differentiating between the grades of OSA severity, NT pro BNP compared to the echocardiographic method showed no significant difference (*p* = 0.59—Table 5).

**Table 5:** Comparison between severe OSA patients vs non severe OSA patients.

Variable	Severe OSA (n = 41)	Non-severe OSA (n = 28)	*P* value
RV, mm	42 (39;45)	38 (35;41)	0.001
FAC, %	25 (21;28)	30 (27;32)	0.001
TAPSE, mm	18 (17;19)	20 (18;20 )	0.015
S’ wave, mm	9 (8;11)	10 (9;12)	0.037
RA-RV gradient, mmHg	36 (35;45)	30 (27;35)	0.001
sPAP, mmHg	43 (42;52)	37 (34;42)	0.001
3D RV GLS, %	− 12.3 (− 15.6; − 9)	− 15.3 (− 17.5; − 11.5)	0.011
3D RVEF, %	30 (27;32)	34 (28;41)	0.026
NT pro BNP, pg/ml	340 (250;723)	230 (159;485)	0.059

## Discussion

To the best of our knowledge this is the first comprehensive study using 3D RV speckle tracking echocardiography as a new quantitative method of assessing RV function in OSA patients.

RV function plays a key role in the prognostic of several cardiac and lung conditions^29–31^, but its echocardiographic assessment is often difficult because of the anatomy of the RV^28^. Conventional 2D echocardiographic parameters, like TAPSE, tissue Doppler S′ wave and FAC, offer information regarding global RV function but fail to evaluate important regional alterations in contractility as well as contraction synchronicity. Furthermore, the values of these standard parameters may be influenced by concomitant RV wall motion deformities^32^. Therefore, utilization of the novel and advanced imaging of myocardial systolic strain through speckle tracking may be beneficial.

Previous studies have shown that patients with OSA displayed impairment of 2D RV GLS. As Buonauro et al.^33^, described in their study, OSA patients showed important reduction in 2D RV GLS (18.2 ± 2.4%, *p* < 0.001), compared to control group. RV GLS was independently associated with sPAP and OSA severity, in the absence of significant changes of other echocardiographic parameters of RV systolic function, such as TAPSE or FAC. In concordance with these results, Li et al.^34^, described significantly reduced RV lateral strain (LS) and strain rates of RV apical segments in patients with mild OSA compared with healthy controls. Hammerstingl et al.^35^, found that RV 2D GLS was significantly impaired in patients with higher AHI, and was positively correlated with the severity of OSA. Furthermore, conventional echocardiography parameters, like TAPSE, S’ wave and FAC were not significantly reduced in these subjects, assuming that they are not sensitive enough in detecting RV subclinical dysfunction.

The last decade brought in significant advancements in the, 3D imaging of the left ventricle (LV), but as stated by Reichek^36^, RV speckle tracking imaging has fallen back in this matter. As Blanchard and DeMaria^37^, mentioned in their review article on RV 3D strain in pulmonary hypertension, RV has a thin wall, making endocardial and epicardial borders difficult to track, moreover the software packages for 3D strain analysis available now are designed for the left ventricle.

Our results suggest that 3D RV GLS is a reliable echocardiographic method with higher specificity in predicting OSA, when compared to the standard RV function parameters. Moreover, we have found that NT pro BNP, a known important biomarker of cardiac stress and implicitly of LV and RV systolic dysfunction is not equally sensitive in predicting RV dysfunction in OSA patients compared to 3D RV GLS.

All 2D and 3D echocardiographic variables assessed in the study were significantly reduced in OSA patients compared to the controls, with strong correlations between FAC, sPAP and 3D RV GLS, and with lesser, but still significant association for S’ wave and TAPSE. There are already several studies in literature demonstrating reduced 2D echocardiographic parameters in OSA population^9,38–40^, evaluation of the role of 3D specific parameters in this pathology, being in progress^41,42^. The superiority of 3D RV strain over 2D RV strain in RV function assessment against cardiac magnetic resonance imaging (MRI) was already demonstrated in a study population with a wide variety of cardiovascular pathologies^43^.

Regarding the presence of sPAP, we obtained in the ROC analysis that a cutoff of > 30 mmHg has a high specificity in predicting the disease. The question arising in this case is if the evaluation of pulmonary artery systolic pressure, could really be a reliable parameter for predicting OSA, considering that in our study, as mentioned before, there were also patients diagnosed with OSA without the presence of PHT, situation in which 3D RV GLS demonstrated a more confident parameter to assess. These findings are supported by the study of Smith et al.^25^, who have shown that in a subset of PHT patients, sPAP correlated weakly with RV longitudinal strain (LS). Extrapolating to our study, the lower values of 3D RV GLS in OSA patients are mainly due to the disease itself and in a less extent due to the presence of PHT. Similar results were found by Altekin et al.^44^. In their study, they used the STE method to assess the presence of right ventricle dysfunction before the occurrence of RV failure and PHT in OSA patients. They concluded that strain could be a reliable method to detect subclinical right ventricle dysfunction among patients with OSA, even in the absence of PHT. On the other hand, Li et al.^20^ demonstrated in their study that 2D and 3D LS are the variables that correlate better with severity of PHT, demonstrating clinical superiority over circumferential strain, radial strain and conventional RV function parameters.

It is well known that OSA is most common in people who are overweight or obese. Obesity is an important anatomical factor, but BMI is not perfect to classify the obese OSA patients because the main factor is fat deposit in the upper airway and abdominal fat^45^. Right ventricular (RV) remodelling in obesity is less well studied than LV remodelling, but it has been reported to include subtle RV cavity dilatation and RV hypertrophy^46^. Sokmen et al. have demonstrated in their study that isolated obesity in young adults was associated with subclinical abnormalities in RV structure and function. It is important to mention that they did not perform a sleep study to determine the contribution of OSA to RV changes ^47^. Correspondingly, in another study evaluating RV function and structure by echocardiography, RV mass and volume was shown to be increased in obese subjects^46^. On the other hand, some studies found no relation between obesity and RV function or structure^48,49^. For example, Yildirimturk et al. showed no changes in the RV functions in obese and overweight who were otherwise healthy subjects. There was no correlation found between the BMI and the RV diameters or functions^49^. Subsequently, approximately 20% of adults with OSA are non-obese. Studies show that this category of OSA patients are four times more likely to develop hypertension than obese without OSA^50^. Non-obese patients are at risk for early atherosclerosis approximately 2.7 times more than obese subjects without OSA^51^. In our study, even though the BMI values in both groups indicate at least overweight, there was a significant difference between the OSA and the control group. The fact that the groups were not BMI matched, remain an important limitation of the study.

Our study also defines cutoff values, which may be clinically applicable for identifying patients with obstructive sleep apnea and for better characterization of disease severity. There are no extensive studies or indexing to sonomicrometry for the classification of normal RV strain values. Alternatively, normal 2D LS is obtained from a five-study meta-analysis of healthy controls^52^ As far as we know a validated value of 3D RV GLS in OSA population has not yet been established,therefore our results may be taken into consideration in future research. We found that lower strain values below the cutoff for RV GLS were associated with the positive diagnosis of OSA, with higher sensitivity and specificity than standard day to day 2D parameters. Especially the cutoff value of 3D RV GLS has an important value, considering that, as far as we know, there is not a standard value validated in OSA. In recent literature there have been proven cutoff values for 3D RV strain vectors in patients with PHT, proven to be highly important for mortality risk assessment^25^. As mentioned before, we have used for the 3D ST evaluation of RV, a software designed for the LV. Being originally created for the evaluation of LV, segmentation is rather limiting for use in RV. It is difficult to delineate the exact location of the septal free wall edge with regard to the fixed segments, which makes it challenging to separate segments from each other. Moreover, the septum is composed of the same fibers as those found in the LV and has to deal not only with loading conditions in the RV but as well with the higher LV afterload^17^. On that account, like the authors of other published studies of RV strain^53–56^, we decided to include the septum in the strain analysis, as a part of the RV, because its shortening plays a part in the ejection phase of the RV and any changes in its contractility reduces the RV performance^57^. On the contrary, there are studies which excluded the septum segments from the analysis based on the fact that ventricular septum is composed of left and right components, which makes it hard for the echocardiography to differentiate^25,58^. Consequently, inclusion of septum will include LV strain values. Extremely, impaired septal strain in patients with associated LV failure, might contribute to the reduction in global RV strain^25^. It is important to mention that, in our study, patients with LV systolic dysfunction were not included, all participants having LVEF above normal.

Furthermore, in our research we also evaluated the role of 3D RV strain in predicting OSA severity compared to standard 2D parameters. The better association with the severe forms of OSA was demonstrated by FAC. Similarly, 3D RV GLS showed good correlation with the severe OSA cases. In the study of Altekin et al., it was shown that TAPSE and values of tissue Doppler were lower in patients with moderate-sever OSA than in the controls and mild OSA; also, they found out that patients with moderate to severe OSA have lower RV strain and RV systolic strain values than controls^38^. Furthermore, similar to our study, Buoanauro et al., demonstrated that subclinical RV dysfunction associated with severe cases of OSA can be determined via STE, in patients with normal RVEF and TAPSE^33^. Regarding 3D RVEF, some reports described that RVEF is similar in patients with and without OSA^59^, which is in contradiction with our results. In our study, 3D RVEF had lower values in the group of OSA compared to healthy volunteers. Furthermore, the cutoff obtained in the ROC analysis may predict RV dysfunction with a good specificity, but lesser than 3D RV GLS and NT pro BNP. Concerning the capability in distinguishing between the severity grades of OSA, 3D RVEF is correlated with the severe cases, but not as strong as 3D RV GLS. On the contrary there is a study in literature showing a strong association between OSA and RVEF^60^.

A study by Quaife et al.^61^, showed a close association between RVEF and RV wall stress, which is the principal trigger to NT pro BNP secretion in OSA, PHT and others RV conditions. This relationship may explain the utility of the biomarker as a tool in detecting RV systolic dysfunction and consequently, OSA. At a cutoff value of > 136 pg/ml, with a sensitivity of 91.8%, we have demonstrated that NT pro BNP may be used in the positive diagnosis of OSA, but with a lower accuracy than 3D RV GLS. On the other hand, there are published studies which show that NT pro BNP is not elevated in patients with OSA ^62^. These results are sustained by another paper^63^ explaining that BNP levels are not elevated in the study group compared to controls. Kita and coworkers^64^ showed that BNP levels are elevated in patients with severe OSA. These results are in contradiction with those of our study. NT pro BNP is not associated with severe cases of OSA, thus it may not be considered a reliable method to differentiate between the severity of disease.

Comprehensive assessment of each individual's characteristics, as well as monitoring and follow-up, are essential to effective management of OSA. Continuous positive air pressure (CPAP) therapy is a first-line treatment for patients diagnosed with OSA^65^. It is already known that OSA has a detrimental effect on both RV and LV function. Several studies support that, effective OSA therapy improves RV and LV function in OSA patients^38,66,67^. The positive effect of CPAP therapy was shown by Chu et al. They proved that after six months treatment with CPAP, RV structure and function had significantly improved, including increased RV ejection fraction and improved RV strains, which practically indicated a pathophysiologic association between RV performance and OSA^66^. In another study, Vitarelli et al. showed that 3D RV ejection fraction was lower and RV dyssynchrony was greater in patients with moderate-severe OSA compared with control subjects. Moreover, RV 3D STE abnormalities improved after chronic application of CPAP^67^. Recently Colish and colleagues found RV and right atrial (RA) diameters positively influenced by 12 months of CPAP treatment in a group of 47 OSA patients^68^. In opposition with these studies are the results of Hammerstingl et al. After 6 months of CPAP therapy, they found a significant increase in systolic LV ejection fraction, bur global RV function parameters, TAPSE and interventricular septum diameter, did not change significantly over the treatment period^35^. All in all, in order to prevent permanent impairment of the left and right ventricular structure and function as a result of OSA, early use of CPAP therapy is recommended in all patients with relevant OSA.

## Limitations

Some limitations of our study consist in the initial exclusion of patients with reduced LVEF, history of ischemic disease and cardiopulmonary decompensated disease. Thus, our results may only be applied to populations that are similar to those in our study. Second, the OSA and control group were not BMI matched, even though the BMI values in both groups indicate at least overweight. Third, an important part of strain analysis had to be rejected because of suboptimal image quality. This included subjects with poor apical view, too large RV for the 3D sector or image dropout. Fourth, 3D STE analysis originally designed for the LV was used for RV assessment in our study. The results obtained apply only to the software used in our study and could not be generalized, considering that 3D STE parameters are restricted by the intervendor variability. Fifth, despite the fact that 3D STE has been validated for strain vectors of LV^69^, there has not been a direct validation for use in the RV. 3D STE would gain utility for future use from validation using sonomicrometry or even better cardiac IRM. Sixth, this is a single center study, with a limited number of patients. Future prospective, long term follow up studies will be necessary to determine the meaningful cutoff values of 3D RV GLS for the detection of RV dysfunction in OSA patients and their prognostic role. However, 3D STE has been disclosed as clinically applicable in LV function assessment, therefore, 3D RV STE might be a valuable method to evaluate RV function in OSA.

## Conclusions

In summary, our study it is the first to show that 3D RV strain imaging may have independent diagnostic and assessment value in patients with OSA. Moreover, 3D RV GLS is a reliable method of assessing the RV global function in OSA, because it correlates well with other established measurements of RV systolic function, like TAPSE, S′ wave and FAC. Furthermore, 3D RV GLS was shown to be a more reliable and accurate parameter in identifying severe cases of OSA, while NT pro BNP showed no association. The role of 3D STE in OSA patients, should be addressed by further prospective studies, as current data yield few studies. If such studies do demonstrate predictive value, 3D strain could eventually become a standard part of the RV echocardiographic exam, much like TAPSE and S’ wave are today.

## Data Availability

The datasets generated and/or analysed during the current study are not publicly available due the fact that they constitute and excerpt of research in progress but are available from the corresponding author on reasonable request.
